# Enhancing Colorimetric Detection of Nucleic Acids on Nitrocellulose Membranes: Cutting-Edge Applications in Diagnostics and Forensics

**DOI:** 10.3390/bios14090430

**Published:** 2024-09-05

**Authors:** Nidhi Subhashini, Yannick Kerler, Marcus M. Menger, Olga Böhm, Judith Witte, Christian Stadler, Alexander Griberman

**Affiliations:** 1 SERATEC Gesellschaft für Biotechnologie mbH, Ernst-Ruhstrat-Str. 5, 37079 Goettingen, Germany; 2 Fraunhofer Institute for Cell Therapy and Immunology (IZI), Branch Bioanalytics and Bioprocesses (IZI-BB), Am Mühlenberg 13, 14476 Potsdam, Germany; 3 Institute for Biochemistry and Biology, University of Potsdam, D-14476 Potsdam, Germany; 4 Sartorius Stedim Biotech GmbH, August-Spindler-Str. 11, 37079 Goettingen, Germany

**Keywords:** lateral flow assay, multiplexing, nucleic acid lateral flow assay (NALFA), molecular beacon, protein-free, amplification-free, body-fluid identification

## Abstract

This study re-introduces a protein-free rapid test method for nucleic acids on paper based lateral flow assays utilizing special multichannel nitrocellulose membranes and DNA-Gold conjugates, achieving significantly enhanced sensitivity, easier protocols, reduced time of detection, reduced costs of production and advanced multiplexing possibilities. A protein-free nucleic acid-based lateral flow assay (NALFA) with a limit of detection of 1 pmol of DNA is shown for the first time. The total production duration of such an assay was successfully reduced from the currently known several days to just a few hours. The simplification and acceleration of the protocol make the method more accessible and practical for various applications. The developed method supports multiplexing, enabling the simultaneous detection of up to six DNA targets. This multiplexing capability is a significant improvement over traditional line tests and offers more comprehensive diagnostic potential in a single assay. The approach significantly reduces the run time compared to traditional line tests, which enhances the efficiency of diagnostic procedures. The protein-free aspect of this assay minimizes the prevalent complications of cross-reactivity in immunoassays especially in cases of multiplexing. It is also demonstrated that the NALFA developed in this study is amplification-free and hence does not rely on specialized technicians, nor does it involve labour-intensive steps like DNA extraction and PCR processes. Overall, this study presents a robust, efficient, and highly sensitive platform for DNA or RNA detection, addressing several limitations of current methods documented in the literature. The advancements in sensitivity, cost reduction, production time, and multiplexing capabilities mark a substantial improvement, holding great potential for various applications in diagnostics, forensics, and molecular biology.

## 1. Introduction

The history of nucleic acid detection can be traced back to the seminal work of researchers who laid the foundation for understanding deoxyribonucleic acid (DNA) and ribonucleic acid (RNA) structure and function. The discovery of the double helix structure of DNA by Watson and Crick in 1953 marked a watershed moment in biology, paving the way for the development of numerous techniques to detect and analyse nucleic acids [1,2]. Early methods such as Southern blotting [3], polymerase chain reaction (PCR) [4], and fluorescence in situ hybridization (FISH) [5] revolutionized molecular biology by enabling scientists to visualize and manipulate nucleic acids with unprecedented precision.

Nitrocellulose membranes emerged as a key substrate for nucleic acid detection in the late 20th century, particularly for lateral flow assay (LFA) developments. Nitrocellulose membranes offer several advantages that make them well-suited for nucleic acid detection compared to other solid supports like polystyrene. Firstly, their high binding capacity allows efficient immobilization of several biomolecules like nucleic acids, ensuring robust signal generation during detection assays. The consistent pore structure of nitrocellulose membranes facilitates uniform distribution of biomolecules across the surface, minimizing variability and enhancing assay reproducibility. Moreover, nitrocellulose membranes are compatible with a wide range of detection methods, including colorimetric, chemiluminescent, fluorescent, and radioactive labelling techniques, providing flexibility in assay design and implementation [6,7].

In colorimetric detection, nitrocellulose membranes serve as the substrate for capturing nucleic acid targets and visualizing them through enzymatic reactions that produce visible color changes. This approach eliminates the need for sophisticated instrumentation, making it suitable for resource-limited settings and on-site rapid testing. The simplicity and rapid turnaround time, 10–20 minutes (min), of colorimetric assays on nitrocellulose membranes enhance their appeal for applications where the timely detection of nucleic acids is crucial, such as infectious disease diagnostics and forensic investigations [8].

Recent advancements in nucleic acid detection have focused on enhancing the sensitivity, specificity, and usability of colorimetric assays on nitrocellulose membranes [9,10,11]. Researchers have developed novel detection strategies and optimized assay conditions to achieve lower detection limits, improved signal-to-noise ratios, and enhanced reliability [12,13,14,15]. These efforts have expanded the utility of colorimetric assays for detecting a wide range of nucleic acid targets, including pathogens, genetic mutations, and biomarkers associated with disease states [16]. The integration of nanoparticles, enzyme-linked probes, and signal amplification strategies has further enhanced the performance of colorimetric assays on nitrocellulose membranes [17]. Nanoparticles conjugated with nucleic acid probes can increase the binding efficiency and enhance the signal output, thereby improving the assay’s sensitivity and reducing false-negative results [18]. Enzyme-linked detection systems, such as horseradish peroxidase (HRP) or alkaline phosphatase (AP), catalyse chromogenic reactions that produce visible color changes, enabling the qualitative and semi-quantitative analysis of nucleic acids [19,20].

The lateral flow assay is mainly a paper-based diagnostic tool used to detect and quantify analytes in liquid samples, often consisting of complex mixtures. It offers rapid results within 5–30 min, enabling easy handling and making it suitable for various applications due to its low development costs and ease of production. LFAs are commonly utilized [8,17]. NALFAs offer numerous advantages by combining the specificity of nucleic acid recognition with the simplicity and speed of lateral flow technology. Nucleic acids also offer several advantages over antibodies, including greater stability in dehydrated form and the capability for site-specific labelling and functionalization through chemical synthesis. These benefits have led to the development of nucleic acid-based LFAs, which include hybridization-based NALFAs (hNALFAs) that utilize the predictable nature of Watson-Crick base pairing for rational design [21,22]. Despite the potential advantages of nucleic acid-based LFAs, traditional methods like sequencing by hybridization to oligonucleotide microchips (SHOM) [23,24] and real-time polymerase chain reaction (RT-PCR) [25,26,27,28] still dominate mainly due to the low sensitivity achieved with colloidal gold conjugation or the complexity needed for signal enhancement using antibodies or silver deposition.

Alternate methods to the classical competitive or sandwich assay formats [29] hNALFAs also exist, which utilize molecular beacons (MBs), single-stranded hairpin DNAs that change structure in the presence of target nucleic acids [30]. These methods can be less sensitive to experimental conditions but require specific modifications for each target microRNA, increasing costs [22,31]. Javani et al. [21] reported an inexpensive and unique LFA design using unmodified oligonucleotides at capture lines without relying on streptavidin or other affinity proteins. They utilized the structural switch of MBs combined with the base stacking hybridization (BSH) phenomenon, offering high selectivity for target oligonucleotides. However, their limit of detection (LOD) was about 10 pmol, higher than that of similar existing methods. This protein-free strategy was adopted in the current study for further research and optimization of NALFA techniques to improve LOD, sensitivity, and specificity. The optimization process began with electrophoresis mobility shift assay (EMSA) to observe DNA-pair hybridization times, the effect of different temperatures on reaction times, and the role of hybridization strand ratios. Additionally, the specificity of DNA molecules with their complementary sequences was confirmed. The NALFA production time was also optimized by refining the intermediate steps of Gold-DNA conjugation. The use of a different dispensing system and uniquely designed membranes allowed multiplexing up to six different target nucleic acid sequences on the same test. In this study, a multiplex test for four targets was developed, demonstrating very high specificity and sensitivity compared to known NALFAs. The advantages of these enhancements are significant. Increased sensitivity allows for detecting lower concentrations of DNA, making the assay suitable for early diagnosis and low-abundance targets. Reduced detection time enhances throughput and efficiency, which is critical for high-demand scenarios like pandemic responses. High specificity together with multiplexing features ensures accurate results, minimizing false positives and false negatives while increasing the confidence level of the rapid tests. These improvements make the assay more reliable, cost-effective, and practical for widespread use.

## 2. Materials and Methods

### 2.1. Materials and Reagents

**Figure 1:**
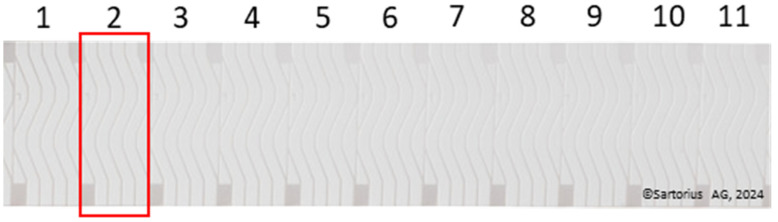
Unisart StructSure^®^ 4-Channel S-Shape nitrocellulose membrane on PET-backing showing 1–11 individual units of StructSure membrane. The red square shows what will be referred to as an individual “StructSure” in the remaining text.

All reagents required for this study were obtained from Carl Roth GmbH (Karlsruhe, Germany). Gold nanoparticles (20 nm citrate-stabilized) were purchased from Nano Flow (Seraing, Belgium). Nitrocellulose membrane (Unisart CN-140, Polyester backing) was purchased from Sartorius Stedim Biotech GmbH (Goettingen, Germany). Unisart StructSure^®^ 4-Channel S-Shape nitrocellulose membranes (3UN14ER084S01WS, Sartorius AG, Goettingen, Germany) were provided by Sartorius AG (Figure 1).

### 2.2. Oligonucleotides

DNA Oligonucleotides (oligos) were designed based on Javani et al. [21] except for the target sequences Tgt-Seq3, Tgt-Seq4 and their corresponding molecular beacons. Oligos were purchased from biomers.net GmbH (Ulm, Germany) and synthesized by standard solid-phase DNA synthesis, followed by purification by HPLC and quality control (QC) by PAGE (Supplement Table S1). The oligos were purchased unmodified and modified at the 5′-end of the DNA oligos. Ctrl-Seq and Molecular beacons (MB1–4) were modified with a 5′-Amino group (NH_2_), and Detect-Seq was modified with a 5′-Thiol group (HS) or 5′-Cy5 dye.

### 2.3. EMSA

To verify hybridization of DNA molecular beacon MB1, MB2, MB3, and MB4, as well as control line oligo Ctrl-Seq with target oligo (Tgt-Seq1–4) and detection oligo (Detect-Seq), an electrophoretic mobility band shift assay (EMSA), as previously described [32], was carried out in a modified form, with modifications as follows. For the analysis of the hybridization of MB with Tgt-Seq and Detect-Seq, 5 pmol of MB was incubated with 20 pmol Tgt-Seq (with a ratio of 1:4) and 10 pmol Detect-Seq (with a ratio of 1:2) in a total volume of 10 µL phosphate-buffered saline (PBS; pH 7.4) for 60 min at 350 rpm and 23 °C on a thermoshaker. The same procedure was performed with 5 pmol of Ctrl-Seq incubated with 10 pmol (with a ratio of 1:2) additionally to MB without either Tgt-Seq or Detect-Seq. As controls, all oligos were carried on individually. The EMSA gels were stained with GelStarTM Nucleic Acid Gel Stain, 10,000x and samples were visualized with a gel documentation system. For optimization of parameters, the ratios of MB1 and 2 to Tgt-Seq1 and 2 and to Detect-Seq were varied (1:1, 1:2, 1:3, 1:4), as well as the incubation time (60 min, 30 min, 15 min, 5 min). A 5′-Cy5-modified Detect-Seq was also used for the performance, which was visualized under the specific wavelength in a gel documentation system.

### 2.4. DNA Immobilization on Gold Nanoparticles

Thiolated DNA oligonucleotides were used for DNA immobilization onto gold nanoparticles (AuNPs). The immobilization was facilitated through the strong affinity between gold and sulfur atoms in the thiol groups. This method ensures stable and covalent attachment of DNA to the AuNPs, which is essential for consistent and reproducible assay performance. A pH-assisted gold immobilization technique using a pH 3.0 citrate buffer was adopted for this study [33]. At first, 500 µL of 0.5 nM AuNPs were mixed with 30 µL of 100 µM thiolated DNA and incubated for 30 min. Then, 10 µL of 500 mM pH 3 Citrate. HCl buffer was then added to enhance the DNA adsorption for 5 min. The solution mixture was then centrifuged at 12,000× *g* at 4 °C for 15 min. The supernatant was discarded, and the pellet was resuspended in 500 µL of PBS. This method significantly accelerated the attachment of thiolated DNA to AuNPs, completing the process in min. It allowed for precise and quantitative DNA adsorption, facilitating the attachment of multiple DNA sequences at predetermined ratios without the need for further quantification. The method was optimized for our needs, and the ratio of DNA to AuNPs was adjusted for the best intensities. For this study a DNA/AuNP ratio of 100 was used, for 0.5 nM AuNPs with a final citrate concentration of 10 mM [34]. UV-Vis spectrophotometry was performed using a DeNovix 31 DS-11+ UV-Vis Spectro-photometer (DeNovix Inc., Wilmington, NC, USA).

### 2.5. LFA Production and Assembly

#### 2.5.1. Lined LFA

Known concentrations of control ssDNA (Ctrl-Seq) and Molecular Beacons (MBs) were dispensed as control line and test line on the strip test using frontline technology of ZX1010 Dispense system (BioDot Limited, Chichester, UK) on nitrocellulose membrane glued on a 30 cm backing card (DCN Dx, Carlsbad, CA, USA). The DNA printed nitrocellulose membranes were then baked in oven at 50 °C for 1 h. The backing cards were then manually assembled using pre-treated conjugate release pads (300 × 10 mm, Grade 6613, Ahlstrom, Espoo, Finland) and absorbent pads (300 × 27 mm, Grade 222, Ahlstrom, Espoo, Finland) on each side of the nitrocellulose membrane creating a sandwich. These 30 cm assembled LFAs were then incised precisely to 6–8 mm wide LFA strips using an automated cutter, Biodot CM5000 Guillotine Cutter (BioDot Limited, Chichester, UK). Then, 10 µL of DNA-AuNPs were manually loaded on conjugate release pads of each strip and LFA was performed in PBS.

#### 2.5.2. Spotted LFA

Control and test spots were deposited on the structured membranes with the precision microdispenser sciFLEXARRAYER S3 pico with a PDC 90 nozzle (SCIENION GmbH, Berlin, Germany).

First, the number of drops required for a visible signal at the control and test spot was determined. The membrane was glued to an adhesive backing card (75 × 350 mm, Kenosha, Amstelveen, Netherlands) prior to spotting. Per StructSure a defined number of NH_2_-Ctrl-Seq (100 pmol/µL) drops was deposited in all four nitrocellulose membrane lanes at the control and test spot positions (Table 1).

**Figure 2:**
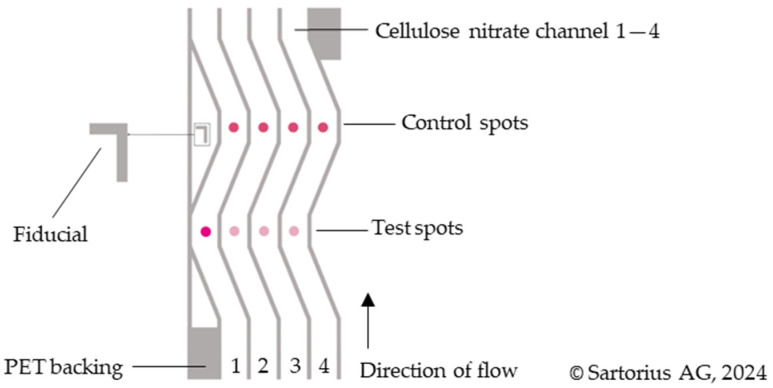
Scheme of the Unisart StructSure^®^ 4-Channel S-Shape membrane. The control spot (CS) in all 4 lanes is spotted with the DNA control Ctrl-Seq (250 pmol/µL). The test spot (TS) is spotted as follows. 1: Ctrl-Seq (250 pmol/µL), 2: MB2 (200 pmol/µL), 3: MB3 (200 pmol/µL), 4: MB4 (200 pmol/µL).

Unisart StructSure^®^ membranes have an L-shaped fiducial (Figure 2). This can be used to automate the spotting and cutting of the assay. In this study, it marks the position of the control spot and is used for orientation.

After spotting, the test strips were dried at 50 °C for 1 h. The assay was assembled with the same adsorbent (300 × 27 mm, Grade 222, Ahlstrom, Espoo, Finland) and release pad mentioned in Section 2.5.1 (300 × 27 mm, Grade 6615, Ahlstrom, Espoo, Finland). The release pad was pre-treated with Tris-buffer (100 mM, pH = 8.0, 0.5% BSA, 0.25% Tween 20) for 3 h and subsequently dried overnight on blotting cardboard at room temperature. Afterwards, the assay was run with 5 µL Detect-Seq-AuNP conjugate in PBS, pH 7.4.

Subsequently, a 4-Channel Unisart StructSure^®^ membrane was glued to backing cards and three of the four nitrocellulose lanes (lanes 2, 3, and 4) were spotted with 50 drops per spot (V_tot_ ~ 21 nL) of different amino-functionalized ssDNA sequences as shown in Figure 2. The first lane (lane 1) was spotted with the control sample in both the control and test spot positions. The test strips were dried at 50 °C for 30 min.

## 3. Results

### 3.1. Verification of Oligonucleotide Hybridization with EMSA

**Figure 3:**
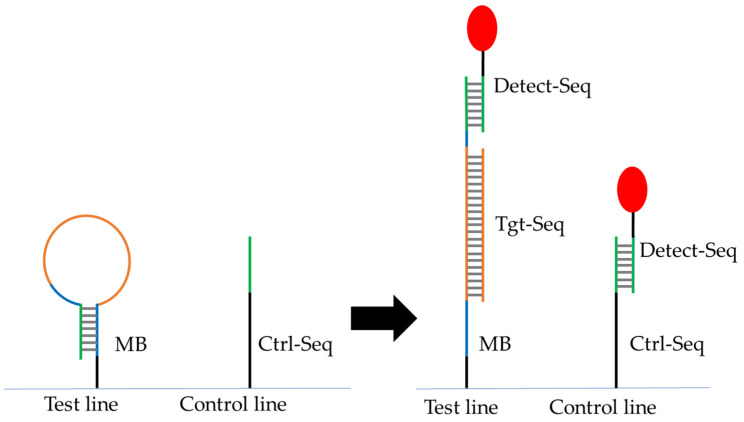
Scheme of the lateral flow assay. Two 5′-NH_2_-modified DNA oligos have been immobilized on the test line (Molecular beacon MB2) and the control line (Ctrl-Seq) on the surface of lateral flow strip. Detect-Seq coupled to a particle or Cy5-dye (red) should always hybridize to Ctrl-Seq but only to MB in the presence of Tgt-Seq in the sample solution. The color codes of segments of sequences represent the color coded nucleotides in Table S1.

In the planned nucleic acid based lateral flow assay (NALFA), MB2 needed immobilization on the test line to hybridize with both the target oligo (Tgt-Seq2) and detection oligo (Detect-Seq) in the sample but not with Detect-Seq alone (Figure 3).

**Figure 4:**
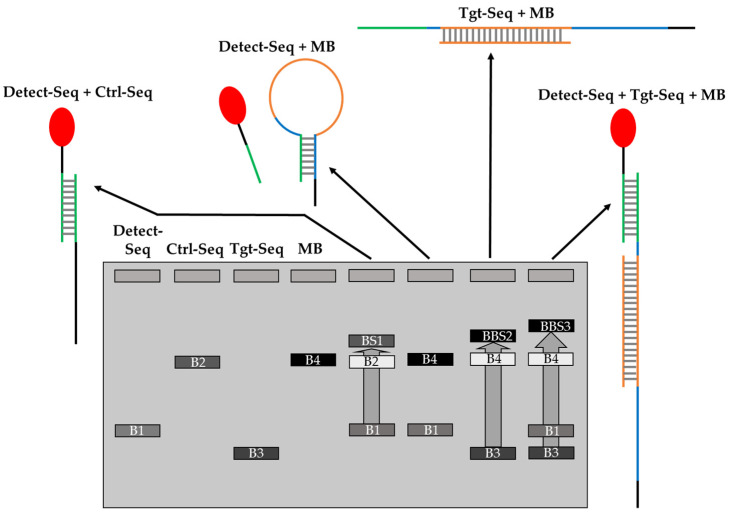
Scheme of hybridization of oligonucleotides MB2 and Ctrl-Seq to Detect-Seq and Tgt-Seq2 by EMSA. B1–4 are the distinctive bands of the oligonucleotides. BS1–3 are the band shifts of Ctrl-Seq with Detect-Seq, MB2 with Tgt-Seq2, as well as MB2 with Tgt-Seq2 and Detect-Seq together. The color codes of segments of sequences represent the color coded nucleotides in Table S1.

To confirm the hybridization of the oligos, EMSA was used to show band shifts (BSs) of the oligo bands when the hybridization of oligos takes place on native polyacrylamide (PAA) gel. Figure 4 shows a scheme with the expected band shifts of the oligos. The Ctrl-Seq band B2 should shift upwards to BS1 when incubated with Detect-Seq, while the MB2 band B4 should not shift upwards when incubated with Detect-Seq (Figure 4). B4 should shift upwards to BS2 when MB2 was incubated only with Tgt-Seq2 and a second time to BS3 when MB2 was incubated with Tgt-Seq2 and Detect-Seq together (Figure 4). When Detect-Seq and Tgt-Seq2 were added in excess, the Detect-Seq band B2 and the Tgt-Seq2 band B3 should still be visible in the incubation mixes with Ctrl-Seq or MB2 (Figure 4).

#### 3.1.1. Band Shift Analysis for Optimizing EMSA Parameters

With an incubation time of 60 min and the ratio 1:4 of MB2 to Tgt-Seq2 and ratio 1:2 of MB2/Ctrl-Seq to Detect-Seq, a band shift BS1 towards the gel pockets was visible with the sample Ctrl-Seq and Detect-Seq without any original B2 (Ctrl-Seq) (Figure S1). No shifting of B4 (MB2) with sample MB2 and Detect-Seq could be seen (Figure S1). MB2 incubated with Tgt-Seq2 showed a band shift of BS2 with a faint band left at the original B4 location (Figure S1). For MB2 with Tgt-Seq2 and Detect-Seq together, a band shift of BS3 was displayed further upwards than BS2, with a faint B4 and slightly stronger than the rest of BS2 (Figure S1).

The parameters of the oligo hybridization were optimized by first testing decreasing incubation time from 60 min to 5 min. MB2 showed the band shift of BS2 with Tgt-Seq2. With decreasing incubation time, the original B4 became slightly stronger (Figure S2). The MB2 sample with Tgt-Seq2 and Detect-Seq together showed the BS3 band shift and a visible BS2 band below (Figure S2). With the decrease in time, BS2 became stronger, as did B4 (Figure S2). The strongest change occurred between an incubation time of 15 min and 5 min (Figure S2).

Furthermore, the hybridization efficiency was investigated by studying the ratios of different oligos. Two parameters were tested: the ratio of MB2 to Tgt-Seq2 and the ratio of MB2 to Detect-Seq. For MB2 to Tgt-Seq2 (1:1 to 1:4), a band shift (BS2) was observed, with the original band (B4) decreasing in intensity as Tgt-Seq2 increased, but BS2 remained strong. For MB2 to Detect-Seq (1:1 to 1:4), a strong BS2 was seen, and with more Detect-Seq, a faint new band appeared above B4. Samples with MB2, Tgt-Seq2, and Detect-Seq showed two band shifts (BS2 and BS3), with BS2 slightly weakening as Detect-Seq increased. The MB1 with Tgt-Seq1 was also optimized with the same parameters and showed similar results (Figure S3).

**Figure 5:**
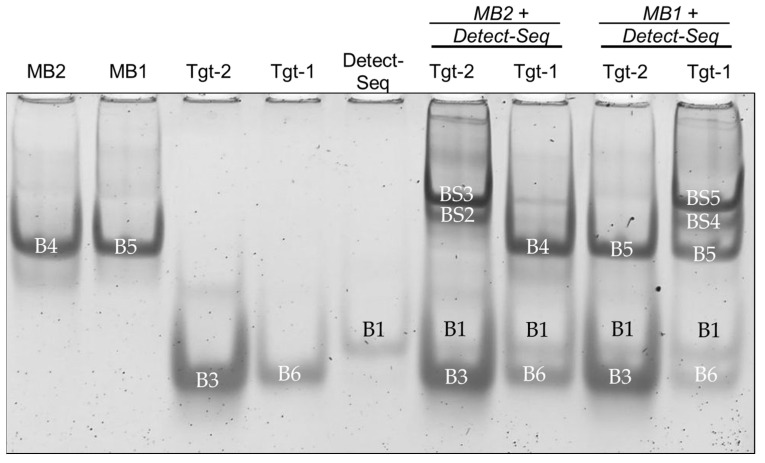
Incubation of oligonucleotides MB1 and MB2 to Detect-Seq and Tgt-Seq1 and 2 by EMSA to evaluate the cross reactivity. MB2 (B4) showed band shifts BS2 and BS3 only with Tgt-Seq2 (B3) and Detect-Seq (B1) not with Tgt-Seq1 (B6). MB1 (B5) showed band shifts BS4 and BS5 only with Tgt-Seq1 (B6) and Detect-Seq (B1) not with Tgt-Seq2 (B3) as well as a still strong B5. MB1/2 = Molecular Beacon, Tgt-Seq1/2 = target oligonucleotide, Detect-Seq = detection oligonucleotide. Incubation time 15 min, ratio 1:3 of MB2 to Tgt-Seq2, ratio 1:2 of MB2/Ctrl-Seq to Cy5-Detect-Seq. EMSA gel stained with GelStarTM Nucleic Acid Gel Stain, 10,000x.

The cross reactivity between MB2 with Tgt-Seq1 and MB1 with Tgt-Seq2 was also checked in EMSA. While the sample of MB2 incubated with Detect-Seq and Tgt-Seq2 showed the shifts BS2 and BS3, when MB2 was incubated with Detect-Seq and Tgt-Seq1, only the B4 without shift was visible (Figure 5). Correspondingly, MB1 incubated with Detect-Seq and Tgt-Seq2 presented no shift; only band B5 did (Figure 5). With Tgt-Seq1, the shifts BS4, BS5, and B5 were detected (Figure 5).

After testing MB1 and MB2, two further MBs were evaluated through EMSA. In the EMSA gel of the MB3 system, the MB3 itself has been visible as band B8, Tgt-Seq3 as band B7 and the Detect-Seq as band B1 (Figure S5a). The MB3 band B8 showed no shift when incubated with Detect-Seq (Figure S5a). A shift was detected when the MB3 was incubated with Tgt-Seq3 above the B8 at BS6, while B8 showed a strong leftover band (Figure S5a). In the last sample with MB3 incubated with Tgt-Seq3 and Detect-Seq, a new shift of BS7 above BS6 emerged, again with a strong leftover band at B8 (Figure S5a). The EMSA gel of the MB4 system showed the MB4 band B10, the Tgt-Seq4 band B9, and the Detect-Seq band B1 (Figure S5b). The incubation of MB4 with Detect-Seq did not lead to a shift of B8 and B1 (Figure S5b). When MB4 was incubated with Tgt-Seq4, two band shifts, BS8 and the even higher BS9, were visible (Figure S5b). The incubation of MB4 with Tgt-Seq4 and Detect-Seq is displayed as a shift BS10, while the shifts BS8 and BS9 showed only faint rest bands (Figure S5b).

#### 3.1.2. Fluorescence Monitoring of Band Shifts in EMSA

**Figure 6:**
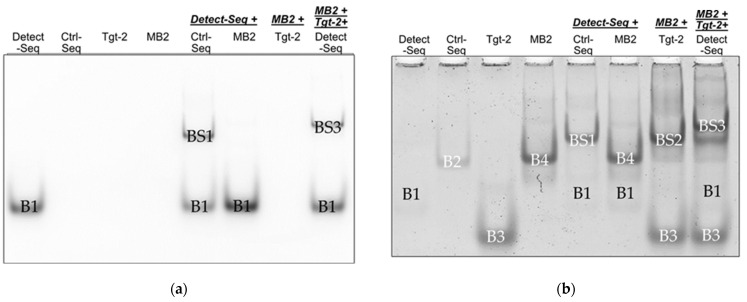
Incubation of oligonucleotides MB2 and Ctrl-Seq to Cy5-Detect-Seq and Tgt-Seq2 by EMSA. (**a**) Cy5 imaging of the EMSA gel where only the Cy5-labeled Detect-Seq (B1) or their band shifts (BS1 and BS3) have been visible; (**b**) EMSA gel stained with GelStarTM Nucleic Acid Gel Stain, 10,000x. MB2 (B4) showed a band shift BS2 only with Tgt-Seq2 (B3) and the additional higher upwards band shift BS3 with Tgt-Seq2 and Cy5-labeled Detect-Seq together. Ctrl-Seq band B2 shifted also to BS1 with Cy5-labeled Detect-Seq. MB2 = Molecular Beacon, Ctrl-Seq = control line oligonucleotide, Tgt-Seq2 = target oligonucleotide, Detect-Seq = 5′ Cy5-labeled detection oligonucleotide. Incubation time 15 min, ratio 1:3 of MB2 to Tgt-Seq2, ratio 1:2 of MB2/Ctrl-Seq to Cy5-Detect-Seq.

The final implementation of all optimized parameters was also tested in EMSA, this time with 5′-Cy5 modified Detect-Seq to additionally monitor the band shift behavior by fluorescence before staining with GelStarTM Nucleic Acid Gel Stain. In the Cy5 image, the Cy5-Detect-Seq shows the band B1, and after incubation with Ctrl-Seq, a band shift BS1 appears on the gel (Figure 6a). No band shift was visible by the incubation of Cy5-Detect-Seq with MB2, but together with MB2 and Tgt-Seq2, the band shift BS3 is shown (Figure 6a). After staining, the distinctive bands B1-B4 of Detect-Seq, Ctrl-Seq, Tgt-Seq2, and MB2 were visible, but B1 was only very faint. The band shift BS1 of sample Cy5-Detect-Seq with Ctrl-Seq could be seen, and the band shifts BS2 of MB2 with Tgt-Seq2 (Figure 6b). Sample MB2 incubated with Tgt-Seq2 and Cy5-Detect-Seq together showed the strong band shift BS3 and weaker band shift BS2 (Figure 6b).

### 3.2. Protein-Free NALFA Test Performance and Assessment of Limit of Detection

**Figure 7:**
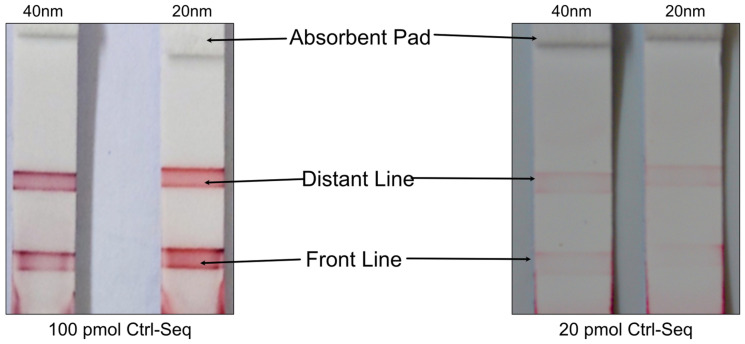
Comparison of 40 nm gold nanoparticles and 20 nm gold nanoparticles for performance in a lateral flow assay. 100 pmol versus 20 pmol; different distances from the conjugate pad (Direction of flow: bottom to top).

After hybridization times at various temperatures were validated using EMSA, the single-stranded DNA (ssDNA) pairs (molecular beacons and target sequences) were tested on lined lateral flow strips. Initially, DNA was immobilized on AuNPs using different published procedures [33,35]. The salt-aging method [35] took 5–6 days to obtain DNA-functionalized AuNPs. This lengthy procedure often challenged the assessment of the stability of gold versus the efficiency of the procedure. Therefore, the salt-aging method was replaced with the pH-assisted method for DNA immobilization on AuNPs [33,34] and was optimized according to the AuNPs and sequences used. The pH-assisted DNA conjugation was completed within 5 min. This extreme improvement encouraged further investigations and optimizations by testing other parameters of gold nanoparticles and their behavior on the NALFA tests. The process began with finding an optimal gold particle size to observe if a significant difference could be observed with the naked eye. Standard lined lateral flow assays and literature-based limit of detection were used for these initial experiments [21]. As demonstrated in Figure 7, no difference in naked-eye detectable intensities was observed between the 40 nm (left side) and 20 nm (right side) gold nanoparticles. Nevertheless, non-uniformity was exhibited by the detection lines, with stronger intensity observed at the edges. This was attributed to the uneven distribution of dispensed DNA, posing a challenge for rectification. In lateral flow immunoassays, the detection line intensity (test and control line) varies with line position. As the distance from the origin of the flow increases, the capillary flow speed decreases [36,37]. Equilibrium in most antigen–antibody reactions is not reached within the time scale of a lateral flow immunoassay but within several hours (h) [38]. Hence, the increasing dwell time of the labeled antibody/antigen at the detection line allows for more antibody/antigen molecules to bind, leading to a more intense signal. However, previous studies primarily focused on antibody detection. Considering the protein-free detection system, which may behave differently, two separate lines were printed with the same DNA molecules at varying distances from the conjugate pad to assess any potential impact on signal intensity. As demonstrated in Figure 7, it was determined that the distance from the conjugate pad does not impact the intensity of the test line (front line) or control line (distant line). Multiplex protein-free strips containing a control line, and two test lines were then developed to explore the feasibility of multiplexing. However, incorporating more than three lines resulted in a decrease in overall signal intensity. Additionally, due to spatial limitations, creating tests with more than two test lines alongside the control line proved challenging and potentially confusing for end users (see Supplement Figure S6). In the experiments, the limit of detection was approximately 20 pmol of DNA, as depicted in Figure 7.

**Figure 8:**
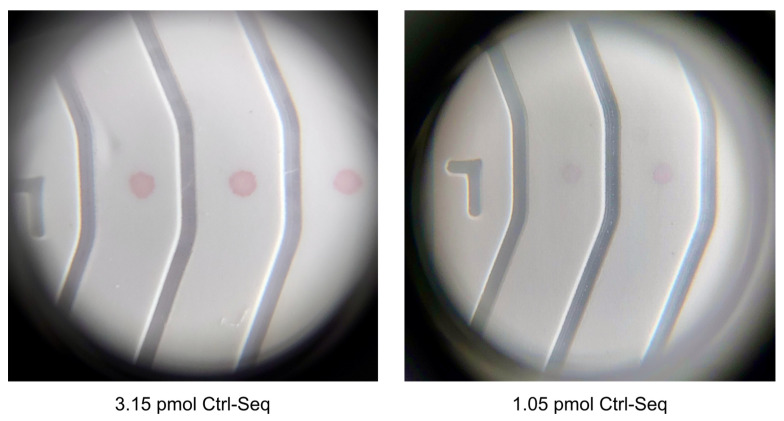
Magnified images (20×) of signal intensities observed on spotted LFA with corresponding amount of DNA on each channel.

The high limit of detection observed initially was not suitable for our research goal of diagnostic and forensic investigations. Consequently, the newly developed Unisart StructSure^®^ membranes were used for further experiments [37]. Additionally, 20 nm AuNPs were selected for subsequent investigations as sharper lines, better contrast, and reduced scattering effects were offered by 20 nm AuNPs compared to 40 nm AuNPs during the preliminary AuNP optimization process and the DNA-conjugation trials. By employing the spotted LFA method with Unisart StructSure^®^ membranes, a detection limit close to 1 pmol of DNA was achieved (Figure 8 and Section 3.4), and multiplexing of up to 6 targets simultaneously on a single strip was allowed by these membranes. However, in the current study, a four-channel membrane was utilized, and the focus was placed on studying only three targets.

### 3.3. Optimization of the DNA-AuNP Conjugation Regarding the DNA to AuNP Ratio

**Figure 9:**
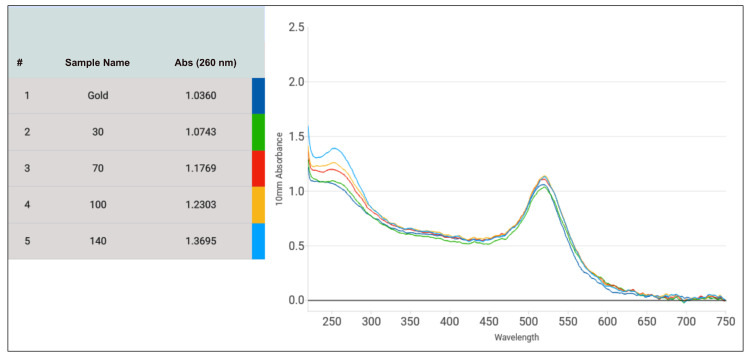
Micro-volume UV-Vis spectra for DNA-AuNP conjugation. Samples 1: AuNPs through conjugation process without DNA, 2: AuNPs conjugated with 30 µL of stock DNA (3 nmol), 3: AuNPs conjugated with 70 µL of stock DNA (7 nmol), 4: AuNPs conjugated with 100 µL of stock DNA (10 nmol), 5: AuNPs conjugated with 140 µL of stock DNA (14 nmol). Peak absorbance measured at 520 nm. The color-coded lines represent the co-coded samples.

An optimized DNA-AuNP conjugation process is also crucial for signal intensity and reproducibility over time and the versatility of experiments. Hence, the ratio of DNA/AuNPs needed for conjugation was further optimized to produce the most intense signal and provide the best stability to the gold for storage. Four different non-linear DNA/AuNPs ratios, approximately 100, 230, 330, and 490, were tested. After conjugation was completed, the microvolume UV-Vis spectra for all five samples containing no DNA or different amounts of DNA were measured. Figure 9 depicts the different amounts of DNA stock (100 µM) in µL added to a total of 500 µL of diluted AuNPs. The 30 µL, 70 µL, 100 µL and 140 µL of stock DNA referred to the DNA/AuNP ratio of 100, 230, 330, and 490, respectively. From the UV-vis spectra obtained, it was evident that with increasing DNA concentration, the gold particles were more stabilized, as observed from increased peak absorbance, while successful conjugation was exhibited with the widening of peaks (Figure 9). Absorbance at 260 nm continuously increased from 1.03 (without any DNA) to 1.07 (30 µL), 1.17 (70 µL), 1.23 (100 µL), and 1.37 (140 µL) also confirmed the increased amount of DNA conjugated to gold with increasing DNA/AuNP ratios. Lowering the DNA/AuNP ratio further from 100 resulted in unstable AuNPs, which frequently changed from deep red color to light purple.

**Figure 10:**
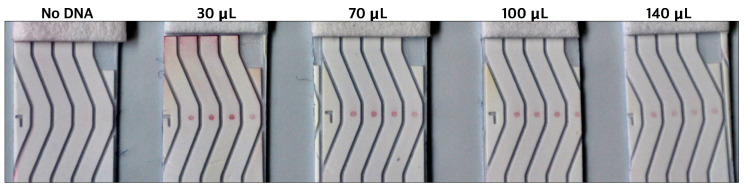
DNA-AuNP intensities on spotted LFA as observed by naked eye in correspondence to the amount of detection DNA (Detect-Seq) used during conjugation. (Direction of flow: bottom to top).

The performance of all five samples of DNA-AuNP conjugates was then tested on our NALFA test strips. It was observed that the AuNPs undergoing the conjugation process without any DNA did not generate any signal, hence proving no AuNP-Ctrl-Seq interaction. All the DNA-conjugated AuNPs generated signals, which further confirmed the successful DNA immobilization of the AuNPs. The intensity of the visual signal observed was inversely proportional to the DNA/AuNP ratio. The higher the concentration of DNA during conjugation, the weaker the signal observed on the NALFA test strips (Figure 10). Thus, the DNA/AuNP ratio of 100, although the least stable among the four ratios, produced the most intense signal. This observation was correlated with the fact that higher concentrations of DNA on AuNPs produce weaker signals due to potential steric hindrance and aggregation, which reduce the availability of binding sites and the efficiency of signal generation. Based on these findings, the DNA/AuNP ratio of 100 was used for subsequent experiments.

### 3.4. Verification of Limit of Detection and Sensitivity

**Figure 11:**
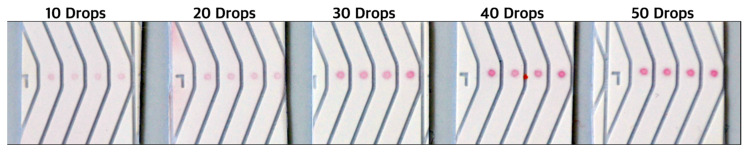
DNA-AuNP intensities visible with naked-eye on spotted NALFA tests as observed by naked eye in correspondence to the amount of detection DNA used during conjugation. The corresponding amount of DNA is outlined in Table 1. (Direction of flow: bottom to top).

The limit of detection in lined NALFA experiments was 20 pmol of Molecular beacon dispensed on nitrocellulose membrane (Figure 7). The new NALFAs were developed with the spotting method, and it was confirmed that a presence of as low as 1.05 pmol of DNA on the strip was sufficient to generate a positive signal with the newly optimized conjugated gold and visible by the naked eye. This is almost a 20 times improvement in the limit of detection and 10 times better than what is known in the literature. As shown in Figure 11, the control spots were faintly visible in LFAs spotted with 10 drops of 420 pL DNA (250 pmol/µL). The signal intensity improved from 10 drops to 40 drops, while increasing further to 50 drops did not show an increase in intensity (Figure 11). With reference to Table 1, it was concluded that 4.2 pmol of DNA was sufficient to generate the maximum intensity on these newly developed tests. Using the information from these experiments, a multiplex NALFA test strip with three different molecular beacons was developed with the molecular beacons spotted at test spots in lanes 2–4 (Section 2.5.2). In these multiplex tests, a minimum of 4.2 pmol of corresponding DNAs was spotted in each lane to maximize the detection efficiency.

### 3.5. Specificity Experiments with Three Different Molecular Beacons and Their Target Sequences

**Figure 12:**
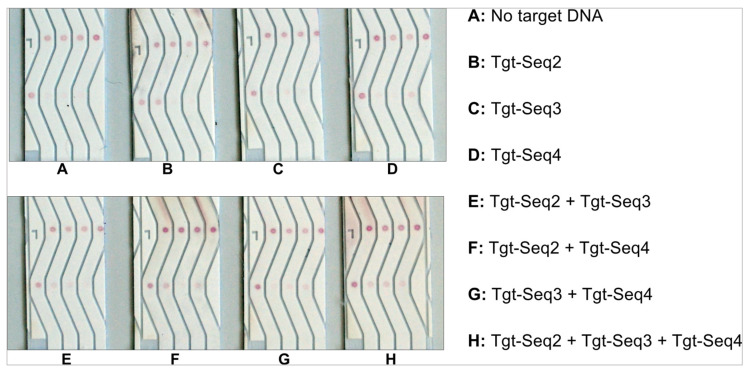
Specificity and cross-reactivity test of the three different molecular beacons (MB2, MB3 and MB4) and their corresponding target sequences (Tgt-Seq2, Tgt-Seq3 and Tgt-Seq4). (Direction of flow: bottom to top).

After optimizing the various conditions for developing a successful NALFA test strip with improved detection limits, it was imperative to substantiate if such a test would be practical for usage against multiple antigens from the same sample in the same test. Hence, we spotted three different test molecular beacons in lanes 2–4, along with the control-Seq in lane 1 (Figure 2). The NALFA strips were then tested with individual target DNAs and different combinations of all three targets: Tgt-Seq2, Tgt-Seq3, and Tgt-Seq4. The Target of the control spots, called Detect-Seq, was conjugated to gold and hence present in all performed tests. For this experiment, 100 pmol of each target sequence (100 pmol/µL) was used. As shown in Figure 12, individual Target sequences Tgt-Seq2 and Tgt-Seq4 were able to develop a test signal without cross-reacting with other molecular beacons within 15 min in B and D, respectively (Figure 12). Tgt-Seq3, however, did not generate a strong signal, which was still clear and specific to its lane. This low signal generated by Tgt-Seq3 can be attributed to the information attained from the EMSA experiments shown in Figure S5a. It was clear from the EMSA results that the hybridization between MB3 and Tgt-Seq3 was weaker, not complete, and only around 50%. Considering the run-time and capillary actions of the developed test, we assume that this amount of time was not enough for the Tgt-Seq3 to hybridize properly with the molecular beacon, and hence, only a weak signal was observed in tests C, E, and G (Figure 12). As expected, in the case of H, all three lanes developed signals showing the individual functionality of all three target sequences, even in the presence of other oligos, hence confirming no cross-reactivity or interaction among the target sequences.

### 3.6. Verification of Functionality with Human Body Fluids

**Figure 13:**
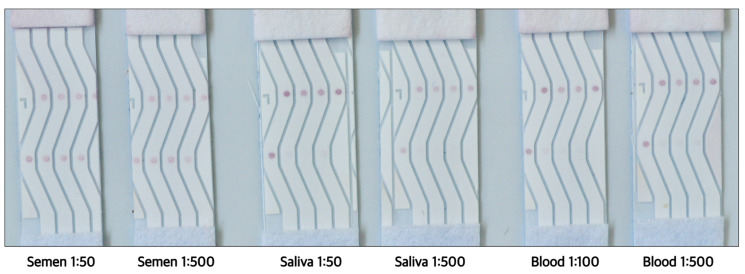
Multiplex NALFA performance with different dilutions of the three body fluids: semen, blood and saliva. With reference to Figure 2, the leftmost lane (Lane 1) had only control spots, the rest of the lanes had different test spots, MB2, MB3, and MB4. All three test spots developed no signal in the presence of saliva or blood. In the case of seminal fluid, all molecular beacons developed strong signals equivalent to the control spot. (Direction of flow: bottom to top).

Next, we tested if such a rapid test would continue to be functional with samples of body fluids and, if yes, up to what dilutions. This information was necessary because human tissue samples are readily diluted for further testing during diagnostic evaluations or forensic analysis. The target sequences in this study were DNA mimics of miRNAs frequently found in seminal fluid but absent in saliva or blood. Therefore, three different body fluids, saliva, seminal fluid, and blood (Lee Biosolutions, Maryland Heights, MO, USA), were tested to verify if the test can correctly identify seminal fluid while not cross-reacting with other body fluids. As shown in Figure 13, the tests confirmed a positive reaction within 2 min for seminal fluid, while saliva and blood consistently showed a negative result. The test showed positive results until the 1:500 dilution for seminal fluid. Saliva showed a negative test without dilution at 1:500 (Figure 13). Blood was diluted to 1:100 and 1:500 due to its deep red color, which can interfere with the test, and, at both dilutions, the NALFA tests were negative (Figure 13).

## 4. Discussion

This study focused on optimizing the parameters of a nucleic acid based lateral flow assay (NALFA) to enhance its sensitivity and specificity for DNA and RNA detection. The optimization process encompassed several crucial steps and testing conditions, such as oligonucleotide hybridization, incubation times, and the selection of materials and specifying optimal protocols for the assay. Furthermore, the present study provides a detailed, step-by-step guide for developing a highly sensitive NALFA from the ground up. These insights empower researchers and manufacturers to effectively leverage their time and resources in perfecting the conditions for their goals and promptly addressing issues early in the process. These advancements have substantial implications across various applications, particularly in diagnostics and forensic analysis.

By employing EMSA to confirm oligo-hybridization, the study illustrated distinct band shifts indicative of successful hybridization on native PAA gel (Figure 6 and Figure S1). For example, Ctrl-Seq band B2 shifted to BS1 when incubated with Detect-Seq, while MB2 did not shift with Detect-Seq alone (Figure 6 and Figure S1). The optimization process involved testing two important parameters: incubation times and oligo ratios. Decreasing the incubation time from 60 to 5 min strengthened the original B4 band of MB2 (Figure S2). Adjusting MB2 to Tgt-Seq2 ratios affected BS2 and B4 band intensities (Figure S3). Varying MB2 to Detect-Seq ratios influenced BS2 and BS3 band visibility and intensity (Figure S4). The selectivity of the MBs to only their corresponding Tgt-Seq could also be shown (Figure 5). These initial insights into the behaviors and distinctions among various oligos during the hybridization process are crucial for planning optimal paper-based lateral flow assay (LFA) protocols and selecting the right materials. Depending on the target nucleic acid sequence, an adaptation of a molecular beacon sequence appears to be necessary in individual cases to achieve complete functionality, as is the case with MB3, for example.

Utilizing the pH-assisted DNA-AuNP conjugation method proved to be critical in improving the quality of functionalized gold nanoparticles and in economizing the production time of the paper-based NALFA. Despite previous studies emphasizing line positioning’s impact on signal intensity in antibody-based systems, this study’s protein-free approach indicated that distance nucleic acid lines from the conjugate pad did not affect signal intensity. This observation is critical for multiplex LFA production but also confirms a different kinetics and equilibrium behavior of nucleic acid interactions as compared to the protein–protein interactions. The initial experiments using standard lined lateral flow assay membranes showed high detection limits of 20 pmol of DNA. Switching to StructSure membranes drastically improved the limit of detection to 1 pmol of DNA, enabling better sensitivity. A non-linear increment approach for the DNA/AuNP ratio was employed optimization because it allows for a more precise determination of optimal conditions and highlights response thresholds. The ratio of 100 showed the best performance in this study, with a significant improvement in signal intensity. However, this ratio is subject to change when one different sequence of DNA is used, or a different type of gold particle is chosen. The optimized LFA demonstrated positive results in detecting the presence of target RNA from body fluids like seminal fluid up to a 1:500 dilution. This showcases the assay’s potential in forensic applications, particularly in identifying bodily fluids at crime scenes. As indicated in Table 2, NALFA provides a valuable balance of sensitivity, speed, and ease of use, particularly suited for point-of-care settings. While PCR and qPCR remain the gold standards in laboratory environments, NALFA’s advantages in rapid diagnostics position it as a strong candidate for widespread implementation.

The improved sensitivity and specificity of the NALFA can be utilized for early detection of diseases by identifying the presence of specific DNA or RNA sequences. The improvements from this study also make these tests compatible with World Health Organization (WHO) recommended ASSURED criteria, which stands for: Affordable, Sensitive, Specific, User-friendly, Robust and rapid, Equipment-free, and delivered to those who need it [40]. This can be particularly useful in point-of-care diagnostics where quick and reliable results are crucial. The ability to detect DNA at very low concentrations and in highly diluted samples makes this method valuable for forensic investigations. It can aid in identifying traces of body fluids at crime scenes, contributing to more accurate and efficient forensic analysis. The high sensitivity of the optimized LFA can be applied to detect environmental contaminants at trace levels. This can be crucial for monitoring water quality and detecting pollutants that may pose health risks. The nucleic acid based LFA can also be used to detect pathogens or genetic modifications in food products. The rapid and sensitive detection can help ensure food safety and compliance with regulatory standards. Researchers can use the LFA to study gene expression and hybridization in various biological samples. The assay’s sensitivity allows for the detection of low-abundance targets, facilitating advanced research in genomics and molecular biology. These findings highlight the efficacy of systematic parameter optimization in NALFA, facilitating the effective refinement of assay conditions to address challenges in molecular diagnostics and forensic sciences.

## 5. Conclusions

Colorimetric detection of nucleic acids on nitrocellulose membranes represents a cornerstone of modern diagnostics and forensic science. These assays combine the robustness of nitrocellulose as a substrate with the simplicity and versatility of colorimetric detection methods, offering rapid, sensitive, and cost-effective solutions for detecting genetic material in diverse biological samples. The optimization of the protein-free NALFA parameters, including hybridization conditions, particle sizes, gold-DNA conjugation method and membrane selection, has significantly enhanced its sensitivity and specificity making this method amplification free for human body fluid identification. The assay was successfully optimized with significantly improved detection limits and demonstrated its applicability for multiplex detection. The experiments revealed that optimal gold nanoparticle size and DNA-to-AuNP ratios were crucial for enhancing signal intensity, achieving a detection limit of 1 pmol, which is a notable improvement compared to conventional methods. By adopting a pH-assisted Gold-DNA conjugation method rather than the prevalent salt-aging method, one week of production time of these NALFAs was saved. Additionally, the multiplexing capability was confirmed by effectively detecting multiple target sequences without cross-reactivity, and the assay demonstrated reliable performance in identifying target microRNA (miRNA) in seminal fluid while maintaining specificity and avoiding false positives from saliva or blood. These advancements broaden the potential applications of this exclusively nucleic acid-based LFA, making it a versatile tool not only in medical diagnostics and forensic analysis but also in environmental monitoring, food safety, and biotechnology research. This study highlights the importance of continuous improvement and adaptation of diagnostic technologies to meet the evolving needs of various fields. Advances in nanotechnology, microfluidics, and bioinformatics are poised to revolutionize nucleic acid detection by improving assay sensitivity, multiplexing capabilities, and integration with digital platforms for data analysis and interpretation. Miniaturization of assay formats and the development of user-friendly devices will expand access to nucleic acid testing in diverse settings, from remote clinics to field-based operations.

## Figures and Tables

**Table 1:** Number of deposited drops with corresponding volumes and amount of DNA dispensed.

No.	Number of Drops Deposited per Spot	Total Volume Deposited per Spot, V_tot_ [nL]	Amount of Ctrl-DNA Deposited [pmol]
1	1	0.42	0.105
2	10	4.2	1.05
3	20	8.4	2.10
4	30	12.6	3.15
5	40	16.8	4.20
6	50	21.0	5.25

**Table 2:** Performance comparison of state-of-the-art nucleic acid-based lateral flow assays (LFAs) with other existing technologies. This table covers key aspects such as sensitivity, specificity, time to result, ease of use, and cost, based on literature [8,13,14,15,17,23,24,25,26,27,28,39]. 1. Sensitivity: Refers to the assay’s ability to detect low concentrations of nucleic acids. 2. Specificity: The ability to correctly identify the target nucleic acid without cross-reacting with non-target sequences. 3. Time to Result: The total time required from sample collection to result. 4. Ease of Use: Refers to how simple it is to perform the test, including sample handling and interpretation. 5. Cost: Includes the cost of consumables, equipment, and overall operational expenses. 6. Equipment Required: Describes the need for specialized equipment. 7. Sample Preparation: Refers to the complexity of preparing samples before testing. 8. Field Applicability: The feasibility of using the technology outside a laboratory setting.

Feature	Nucleic Acid-Based LFA	Antibody Based LFA	PCR (Polymerase Chain Reaction)	qPCR (Quantitative PCR)	CRISPR-Based Assays	Microarrays
**Sensitivity**	High (pM)	Moderate (ng/mL)	Very High (aM)	Very High (aM)	High (pM)	Moderate (ng/mL)
**Specificity**	Very High	High	Very High	Very High	High	High
**Time to Result**	10–20 min	20–30 min	1–3 h	1–3 h	30–60 min	4–8 h
**Ease of Use**	Simple, user-friendly	Simple, user-friendly	Requires specialized equipment	Requires specialized equipment	Simple, user-friendly	Complex, requires specialized equipment
**Cost**	Low to Moderate	Moderate	High	High	Moderate to High	High
**Equipment Required**	Minimal (LFA device)	Minimal (LFA device)	PCR Machine	qPCR Machine	Minimal (LFA device)	Microarray Scanner
**Sample Preparation**	Minimal	Minimal	Moderate to High	Moderate to High	Minimal	High
**Field Applicability**	High	Moderate	Low (lab-based)	Low (lab-based)	Moderate	Low (lab-based)

## Data Availability

The original contributions presented in the study are included in the article/Supplementary Material, further inquiries can be directed to the corresponding author.

## Supplementary Materials

The following supporting information can be downloaded at: https://www.mdpi.com/article/10.3390/bios14090430/s1, Figure S1: Incubation of oligonucleotides MB2 and Ctrl-Seq to Detect-Seq and Tgt-Seq2 by EMSA. All four DNA oligos showed a distinct band (B1-B4). Ctrl-Seq band B2 band shifted strongly to BS1 with Detect-Seq (B1). MB2 (B4) showed no band shift only with Detect-Seq (B1), but the strong band shift BS2 only with Tgt-Seq2 (B3) or the higher upwards band shift BS3 with Detect-Seq in the presence of Tgt-Seq2. MB2 = Molecular Beacon, Ctrl-Seq = control line oligonucleotide, Tgt-Seq2 = target oligonucleotide, Detect-Seq = detection oligonucleotide. Incubation time 60 min, ratio 1:4 of MB2 to Tgt-Seq2, ratio 1:2 of MB2/Ctrl-Seq to Detect-Seq. Stained with GelStarTM Nucleic Acid Gel Stain, 10,000X. Figure S2. Variation of incubation time (60 to 5 min) of oligonucleotides MB2 to Detect-Seq and Tgt-Seq2 by EMSA. DNA oligo MB2 (B4) showed shifting to BS2 with Tgt-Seq2 (B3), and additionally with Detect-Seq (B1) and Tgt-Seq2 together to BS3. The band shifts have become weaker with shorter incubation time. MB2 = Molecular Beacon, Tgt-Seq2 = target oligonucleotide, Detect-Seq = detection oligonucleotide. Ratio 1:4 of MB2 to Tgt-Seq2, ratio 1:2 of MB2 to Detect-Seq. Stained with GelStarTM Nucleic Acid Gel Stain, 10,000X. Figure S3. Variation of molar ratio (1:1 to 1:4) of the oligonucleotides MB2 to Tgt-Seq2 without/with Detect-Seq by EMSA. DNA oligo MB2 (B4) showed the band shift BS2 with Tgt-Seq2 (B3), and additionally the band shift BS3 with Detect-Seq (B1) and Tgt-Seq2 (B3) together. The band shift has become more pronounced with increasing surplus of Tgt-Seq2. MB2 = Molecular Beacon, Tgt-Seq2 = target oligonucleotide, Detect-Seq = detection oligonucleotide. Incubation time 15min, ratio 1:2 of MB2 to Detect-Seq. Stained with GelStarTM Nucleic Acid Gel Stain, 10,000X. Figure S4. Variation of molar ratio (1:1 to 1:4) of the oligonucleotides MB2 to Detect-Seq without/with Tgt-Seq2 by EMSA. MB2 showed band shift BS2 with Tgt-Seq2, and additionally the band shift BS3 with Detect-Seq (B1) and Tgt-Seq2 (B3) together. The band shift has become more pronounced with increasing surplus of Detect-Seq. MB2 = Molecular Beacon, Tgt-Seq2 = target oligonucleotide, Detect-Seq = detection oligonucleotide. Incubation time 15 min, ratio 1:3 of MB2 to Tgt-Seq2. Stained with GelStarTM Nucleic Acid Gel Stain, 10,000X. Figure S5. Incubation of oligonucleotides MB3 and MB4 with Detect-Seq, Tgt-Seq3 and Tgt-Seq4 respectively by EMSA. (a) MB3 (B8) showed band shift BS6 only with Tgt-Seq3 (B7) and the third band shift BS7 with Tgt-Seq3 and Detect-Seq (B1) together. (b) MB4 (B10) showed two band shifts BS8 and BS9 only with Tgt-Seq4 (B9) and the additional higher upwards band shift BS10 with Tgt-Seq4 and Detect-Seq (B1) together. MB3 and 4 = Molecular Beacons, Tgt-Seq 3 and 4 = target oligonucleotides, Detect-Seq = detection oligonucleotide. Incubation time 15 min, ratio 1:3 of MB3 and 4 to Tgt-Seq 3 and 4, ratio 1:2 of MB3 and 4 to Detect-Seq. EMSA gels stained with GelStarTM Nucleic Acid Gel Stain, 10,000X. Figure S6. Multiplex NALFA performance in lined LFA format. (Direction of flow: bottom to top). Table S1. List of Oligonucleotides used in this work. The colour codes of some bases represent parts of the sequences in Figure 3 and Figure 4. PolyA: A10 to A40; N: hidden code of specific bases. Table S1. List of Oligonucleotides used in this work. The colour codes of some bases represent parts of the sequences in Figure 3 and Figure 4. PolyA: A10 to A40; N: hidden code of specific bases.
