# Supplementary material for: Enhancing Colorimetric Detection of Nucleic Acids on Nitrocellulose Membranes: Cutting-Edge Applications in Diagnostics and Forensics
Source: Biosensors (Basel). 2024 Sep 5;14(9):430. doi: 10.3390/bios14090430 (PMC11429540; doi:10.3390/bios14090430)
Supplement: Supplementary file 1 [file biosensors-14-00430-s001.zip › biosensors-3178954-supplementary.pdf]

biosensors

MDPI

Article

Enhancing Colorimetric Detection of Nucleic Acids on Nitrocellulose Membranes: Cutting-Edge Applications in Diagnostics and Forensics

Nidhi Subhashini, Yannick Kerler, Marcus M. Menger, Olga Böhm, Judith Witte, Christian Stadler and Alexander Griberman

Table S1. List of Oligonucleotides used in this work. The colour codes of some bases represent parts of the sequences in Figure 3 and 4. PolyA: A10 to A40; N: hidden code of specific bases.

Name	Sequence 5′ to 3′	Length [nt]
Detect-Seq	PolyAGTAAAACGACGGCCAGT	27
Ctrl-Seq	PolyAACTGGCCGTCGTTTTAC	58
Tgt-Seq1	CAGCAGCAATTCATGTTTTGAA	22
MB1	PolyAGTAAAACGACGGCCA TTCAAAACATGAATT-GCTGCT GACTGGCCGTCGTTTTAC	71
Tgt-Seq2	TGTGCGTGTGACAGCGGCTGA	21
MB2	PolyAGTAAAACGACGGCCA TCAGCCGCTGTCACAC-GCACA GACTGGCCGTCGTTTTAC	70
Tgt-Seq3	AAAGTNNNGCGNNNTTTNNNCGT	23
MB3	PolyAGTAAAACGACGGCCAAC-GNNNAAANNNCGCNNNACTTTGACTGGCCGTCGTTTTAC	72
Tgt-Seq4	TANNNCACNNNGTGNNNGCC	20
MB4	PolyAGTAAAACGACGGCCAGGCNNNCACNN-NGTGNNNTAGACTGGCCGTCGTTTTAC	69

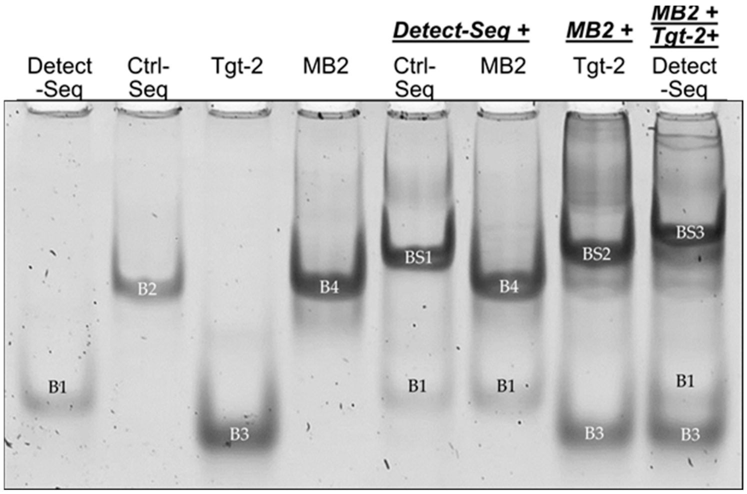

Figure S1. Incubation of oligonucleotides MB2 and Ctrl-Seq to Detect-Seq and Tgt-Seq2 by EMSA. All four DNA oligos showed a distinct band (B1-B4). Ctrl-Seq band B2 band shifted strongly to BS1 with Detect-Seq (B1). MB2 (B4) showed no band shift only with Detect-Seq (B1), but the strong band shift BS2 only with Tgt-Seq2 (B3) or the higher upwards band shift BS3 with Detect-Seq in the presence of Tgt-Seq2. MB2 = Molecular Beacon, Ctrl-Seq = control line oligonucleotide, Tgt-Seq2 = target oligonucleotide, Detect-Seq = detection oligonucleotide. Incubation time 60 min, ratio 1:4 of MB2 to Tgt-Seq2, ratio 1:2 of MB2/Ctrl-Seq to Detect-Seq. Stained with GelStarTM Nucleic Acid Gel Stain, 10,000X.

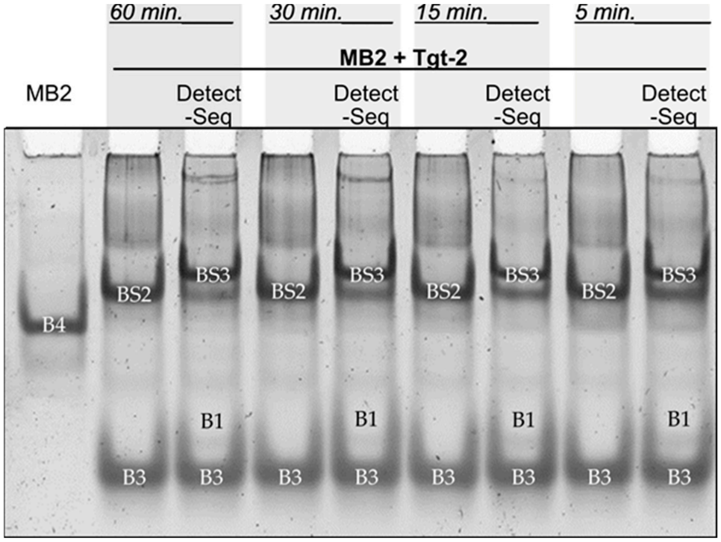

Figure S2. Variation of incubation time (60 to 5 min) of oligonucleotides MB2 to Detect-Seq and Tgt-Seq2 by EMSA. DNA oligo MB2 (B4) showed shifting to BS2 with Tgt-Seq2 (B3), and additionally with Detect-Seq (B1) and Tgt-Seq2 together to BS3. The band shifts have become weaker with shorter incubation time. MB2 = Molecular Beacon, Tgt-Seq2 = target oligonucleotide, Detect-Seq = detection oligonucleotide. Ratio 1:4 of MB2 to Tgt-Seq2, ratio 1:2 of MB2 to Detect-Seq. Stained with GelStarTM Nucleic Acid Gel Stain, 10,000X.

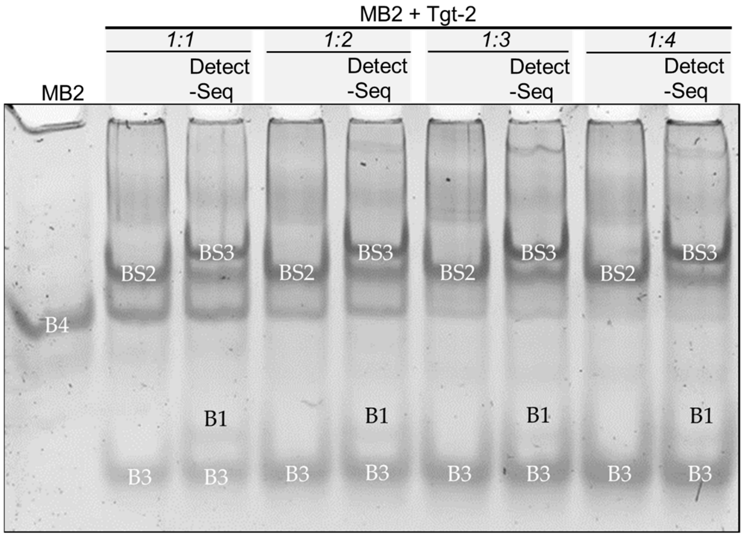

Figure S3. Variation of molar ratio (1:1 to 1:4) of the oligonucleotides MB2 to Tgt-Seq2 without/with Detect-Seq by EMSA. DNA oligo MB2 (B4) showed the band shift BS2 with Tgt-Seq2 (B3), and additionally the band shift BS3 with Detect-Seq (B1) and Tgt-Seq2 (B3) together. The band shift has become more pronounced with increasing surplus of Tgt-Seq2. MB2 = Molecular Beacon, Tgt-Seq2 = target oligonucleotide, Detect-Seq = detection oligonucleotide. Incubation time 15min, ratio 1:2 of MB2 to Detect-Seq. Stained with GelStarTM Nucleic Acid Gel Stain, 10,000X.

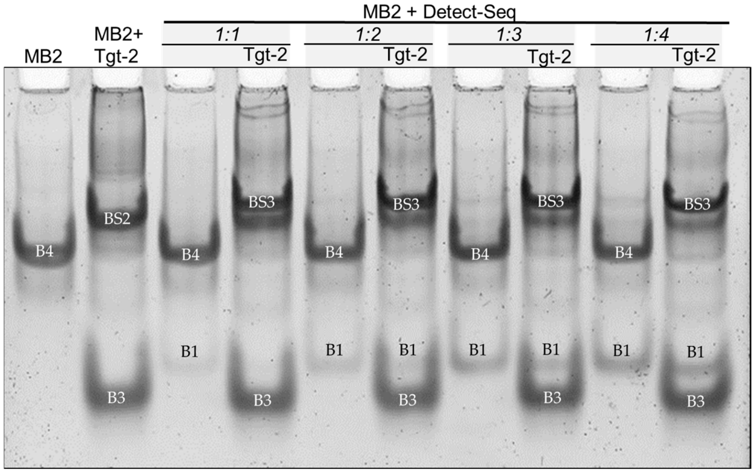

Figure S4. Variation of molar ratio (1:1 to 1:4) of the oligonucleotides MB2 to Detect-Seq without/with Tgt-Seq2 by EMSA. MB2 showed band shift BS2 with Tgt-Seq2, and additionally the band shift BS3 with Detect-Seq (B1) and Tgt-Seq2 (B3) together. The band shift has become more pronounced with increasing surplus of Detect-Seq. MB2 = Molecular Beacon, Tgt-Seq2 = target oligonucleotide, Detect-Seq = detection oligonucleotide. Incubation time 15 min, ratio 1:3 of MB2 to Tgt-Seq2. Stained with GelStarTM Nucleic Acid Gel Stain, 10,000X.

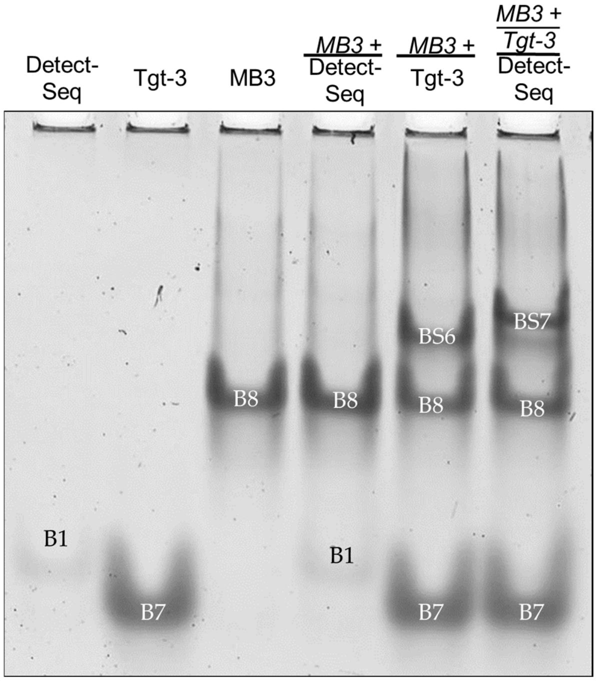

(a)

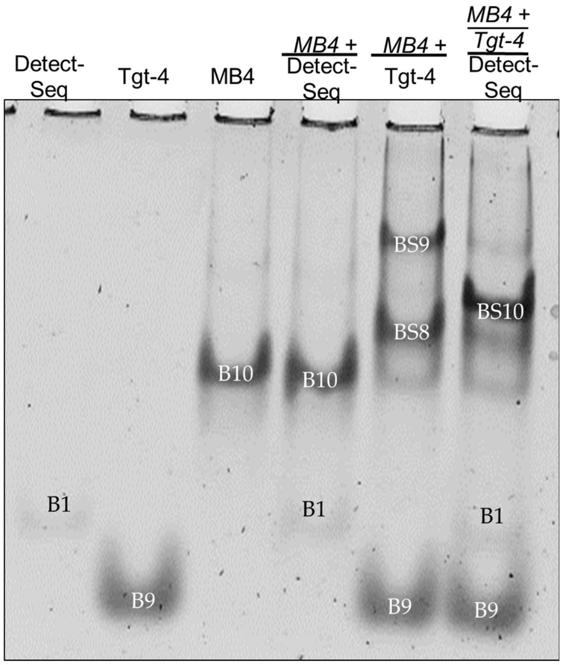

(b)

Figure S5. Incubation of oligonucleotides MB3 and MB4 with Detect-Seq, Tgt-Seq3 and Tgt-Seq4 respectively by EMSA. **(a)** MB3 (B8) showed band shift BS6 only with Tgt-Seq3 (B7) and the third band shift BS7 with Tgt-Seq3 and Detect-Seq (B1) together. (**b**) MB4 (B10) showed two band shifts BS8 and BS9 only with Tgt-Seq4 (B9) and the additional higher upwards band shift BS10 with Tgt-Seq4 and Detect-Seq (B1) together. MB3 and 4 = Molecular Beacons, Tgt-Seq 3 and 4 = target oligonucleotides, Detect-Seq = detection oligonucleotide. Incubation time 15 min, ratio 1:3 of MB3 and 4 to Tgt-Seq 3 and 4, ratio 1:2 of MB3 and 4 to Detect-Seq. EMSA gels stained with GelStarTM Nucleic Acid Gel Stain, 10,000X.

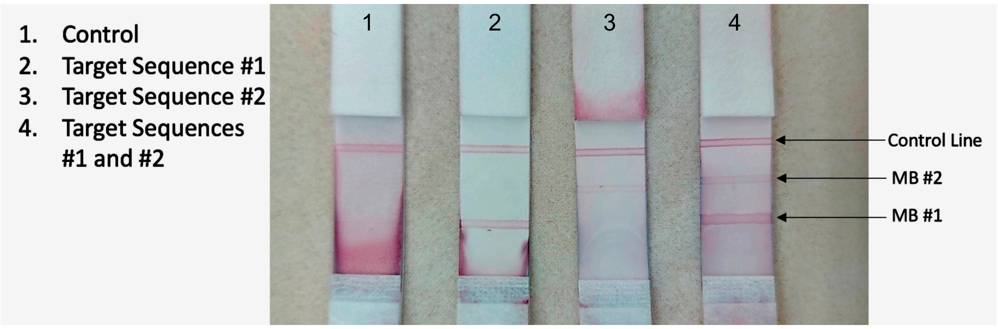

Figure S6. Multiplex NALFA performance in lined LFA format. (Direction of flow: bottom to top).
